# Gut Microbiota in Acute Myeloid Leukemia: From Biomarkers to Interventions

**DOI:** 10.3390/metabo15090568

**Published:** 2025-08-25

**Authors:** Meifen Ji, Meixia Ji, Yebo Zhong, Lewen Shao

**Affiliations:** 1 Department of Nursing, The First Affiliated Hospital, Zhejiang University School of Medicine, Hangzhou 310003, China; y218180133@zju.edu.cn; 2 Lishui People’s Hospital, Lishui 321400, China; zmjmx0416@163.com (M.J.); zhongyebo123@163.com (Y.Z.)

**Keywords:** AML 1, gut microbiota 2, biomarker 3, metabolites 4, FMT 5

## Abstract

Acute myeloid leukemia (AML), the most common acute leukemia among adults, poses significant therapeutic challenges due to diagnostic limitations and the frequent development of treatment resistance. While genomics-based approaches have advanced, DNA aberrations do not always reflect the expression levels of genes and proteins, which are more tightly connected to disease phenotypes. Recently, the role of the gut microbiota in AML has gained increasing attention. AML patients often exhibit gut microbiota dysbiosis, which is linked to disease progression and heightened infection risk. Mounting evidence indicates that gut microbiota metabolism influences hematopoiesis and immune function via the “gut-bone marrow axis,” with microbiota composition and diversity significantly affecting treatment outcomes and prognosis. High-throughput sequencing and metabolomics have identified correlations between gut microbiota composition and its metabolic products with AML clinical characteristics, paving the way for new biomarkers in diagnosis and prognosis. Additionally, treatments such as fecal microbiota transplantation (FMT) show promise in enhancing chemotherapy efficacy and improving patient outcomes. This review highlights recent advances in understanding the role of the gut microbiota in AML and explores new perspectives for its diagnosis and treatment.

## 1. Acute Myeloid Leukemia (AML) and Gut Microbiota

### 1.1. AML

AML is a heterogeneous malignancy characterized by the infiltration of leukemic blasts in the bone marrow. Its pathogenesis involves the accumulation of abnormal alleles in hematopoietic progenitor cells, leading to impaired differentiation, enhanced self-renewal capabilities, and uncontrolled proliferation [1]. Despite the application of novel therapeutic strategies that have improved the 5-year survival rate to approximately 28%, the long-term prognosis for patients remains suboptimal [2]. Particularly, some low-risk patients—especially those in the elderly population—still face poor outcomes despite receiving standard treatment regimens [3,4].

Current clinical management of AML is hampered by inaccurate risk stratification at the time of diagnosis, leading to inappropriate treatment choices. Traditional diagnostic criteria for AML primarily rely on the maturation status of leukemic cells [5], while the most recent WHO classification places greater emphasis on genetic abnormalities, categorizing AML with specific molecular features as distinct subtypes and, in some cases, eliminating the previous threshold of 20% blasts [6]. Prognostic stratification and treatment decisions for AML continue to rely heavily on mutational and cytogenetic features [7,8]. However, the correlation between genomic information and clinical outcomes remains inconsistent, particularly in the intermediate-risk group [9]. Despite advancements, reliable translational biomarkers for prognosis are still lacking [10].

The main treatment modalities for AML include chemotherapy, targeted therapy, and hematopoietic stem cell transplantation (HSCT). Patients frequently exhibit resistance to chemotherapy and/or targeted therapies. Since the establishment of the “7 + 3” regimen in 1973, chemotherapy resistance has remained one of the most significant challenges in AML treatment [11]. Furthermore, while in-depth research into the molecular characteristics of AML has greatly enhanced our understanding of its pathogenesis, effective treatment strategies for relapsed or refractory AML remain limited, highlighting the urgent need to identify new adjunctive therapeutic interventions.

### 1.2. Gut Microbiota in AML

In recent years, the gut microbiota, often referred to as the “second genome,” has increasingly been recognized for its central role in regulating tumor immunity and metabolism. Comprising trillions of microorganisms, this dynamic ecosystem interacts deeply with human physiological functions through its metabolic activities. The gut microbiota occupies a pivotal role in maintaining homeostasis, where these microbial communities engage in a mutually beneficial relationship with the host by modulating immune responses, mucosal barriers, and inhibiting pathogen colonization [12]. Specifically, the gut microbiota plays a crucial role in immune homeostasis, contributing significantly to physiological and pathological processes through the exchange of metabolic products and the regulation of immune stability [13].

Dysbiosis has been demonstrated to have dual pathological implications, serving both as a direct trigger for various diseases and as a biomarker of host immune and metabolic disturbances. Existing studies have clearly elucidated the multi-stage associations between gut microbiota dysbiosis and the development of cardiovascular diseases, neurodegenerative disorders, metabolic syndrome, and malignancies [14]. Notably, recent research has extended these findings to hematologic malignancies, particularly AML, where patients exhibit significant reductions in gut microbiota α-diversity indices. Abnormal abundances of specific microbial taxa have been strongly correlated with disease progression [15].

An increasing body of evidence highlights the association between the human microbiome and the pathogenesis of AML. Numerous studies emphasize the potential link between gut microbiota composition and AML treatment responses, prognosis, and infection-related complications [15,16]. Although the precise relationship between the microbiota and AML pathogenesis remains to be fully elucidated, existing research suggests that microbiota-induced metabolic pathway dysregulation may play a significant role in both the initiation of leukemia and the maintenance of leukemic cells [17]. The study of the human microbiome and its metabolic products provides new perspectives for understanding the biological characteristics of AML.

This review systematically summarizes the latest advancements regarding the role of gut microbiota in the discovery of AML biomarkers and targeted therapies, with a particular focus on the specific mechanisms by which gut microbes and their metabolites influence AML. By reviewing relevant translational medical research, this article highlights the potential clinical value of the gut microbiota, laying a theoretical foundation for diagnostic and therapeutic applications based on microbiota-host interactions. In addition, targeting specific microbiota-related features offers a promising new direction for AML treatment interventions.

## 2. Potential Mechanisms of Gut Microbiota in AML Pathogenesis

**Figure 1:**
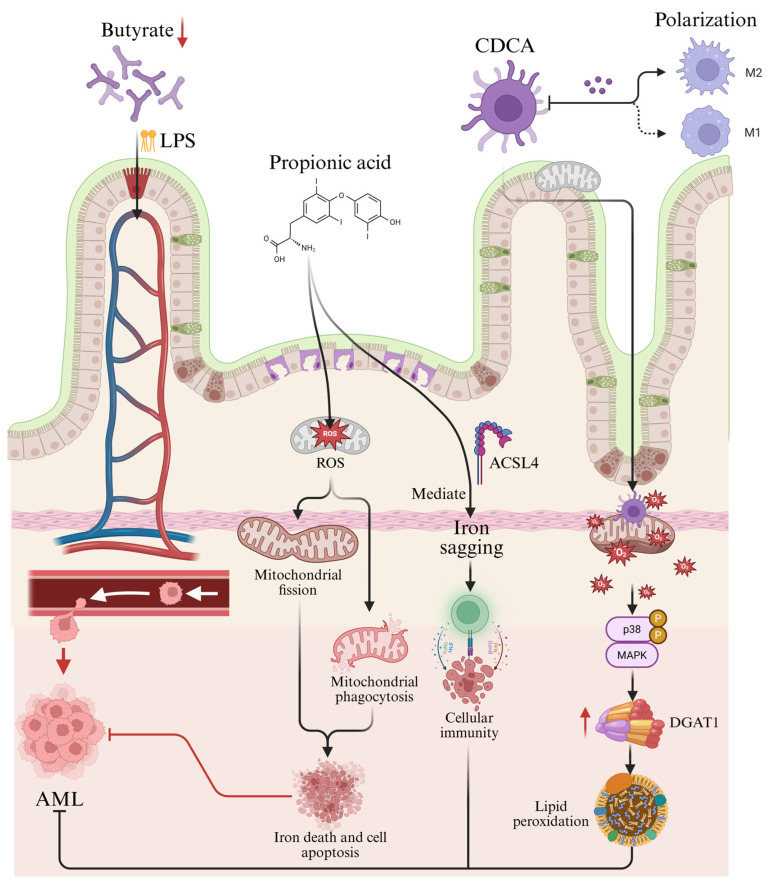
Potential Mechanisms of Gut Microbiota-Derived Metabolites in AML Pathogenesis. How gut microbiota metabolites influence AML: Following gut microbiota dysbiosis, microbial metabolic byproducts translocate across the compromised intestinal barrier into systemic circulation, directly contributing to AML progression. (1) The short-chain fatty acid propionate specifically induces ferroptosis. (2) CDCA inhibits M2 macrophage polarization, thereby blocking M2 macrophage-mediated proliferation. (3) At the molecular level, CDCA interacts with mitochondria, inducing structural abnormalities and excessive ROS production, which ultimately modulates AML progression.

Despite abundant evidence linking the gut microbiota with the pathogenesis and progression of AML, the precise molecular mechanisms underlying this relationship remain incompletely understood (Figure 1).

Growing evidence implicates gut microbiota dysbiosis in AML pathogenesis through specific molecular mechanisms involving microbial metabolite signaling and immune modulation (Figure 1). The depletion of butyrate-producing bacteria (e.g., *Faecalibacterium*), as observed in both AML patients and murine models by Wang et al. [15], reduces intestinal butyrate levels, compromising barrier function through diminished histone deacetylase (HDAC) inhibition and GPR109A receptor activation. The impairment of the intestinal barrier function enhances the translocation of bacterial-derived products, such as lipopolysaccharide (LPS), into systemic circulation, thereby promoting leukemogenic processes [15].

At the metabolite level, gut microbiota-derived short-chain fatty acids (SCFAs) exert direct anti-leukemic effects. Wei et al. [18] demonstrated that propionate induces ferroptosis in AML cells through two coordinated mechanisms: (1) Additionally, propionate can induce iron depletion through the mediation of Acyl-CoA Synthetase Long Chain Family Member 4 (ACSL4); and (2) propionate could promote the accumulation of reactive oxygen species (ROS) and disrupt redox balance, leading to mitochondrial fission and mitophagy, thereby enhancing ferroptosis and apoptosis in AML cells. This dual action culminates in lethal lipid peroxidation characteristic of ferroptosis while simultaneously enhancing AML cell immunogenicity [18].

Chenodeoxycholic acid (CDCA) represents another critical microbial metabolite with multi-targeted effects in AML. As shown by in vitro studies [17], CDCA: (1) suppress M2 macrophage polarization, thereby disrupting this pro-leukemic niche; and (2) directly induces mitochondrial structural abnormalities and ROS overproduction in AML cells. The resulting oxidative stress activates p38 mitogen-activated protein kinase (MAPK) signaling, which upregulates diacylglycerol O-acyltransferase 1 (DGAT1) to drive excessive lipid droplet accumulation and peroxidation—ultimately triggering AML cell death [17].

Elucidating these mechanisms provides a framework for developing microbiota-targeted therapies while highlighting potential biomarkers for AML progression monitoring. Subsequent discussion will explore how these molecular pathways inform therapeutic strategies and prognostic assessment.

## 3. Gut Microbiota in Biomarker Hunting of AML

### 3.1. Gut Microbiota Composition

Approximately 20% of tumors in humans are linked to microbial infections [19]. Increasingly, research has focused on the relationship between gut microbiota diversity, composition, and their metabolites in the pathogenesis of AML. However, data on the specific pathways through which these metabolites change remain relatively scarce. The most extensive studies have concentrated on the multifaceted roles of SCFAs derived from gut microbiota in AML progression (Table 1).

Multiple cohort studies have confirmed that AML patients exhibit characteristic gut microbiota dysbiosis. Compared to healthy controls, AML patients show a significant reduction in both the α diversity index and β diversity of their gut microbiota [15], with particular attention given to the dynamic changes in key functional bacterial genera: the relative abundance of *Faecalibacterium* decreases, playing a crucial role in AML progression. Xu et al. employed 16S rRNA high-throughput sequencing to systematically compare the microbiota profiles of AML patients and healthy individuals [20]. They found that the *Firmicutes*/*Bacteroidetes* ratio was significantly higher in the AML group compared to controls. LEfSe identified *Collinsella* and *Coriobacteriaceae* as specific biomarkers for AML patients. Similarly, Rattanathammethee et al. reached comparable conclusions, showing a notable increase in the relative abundance of *Firmicutes*, which became a dominant genus in AML patients compared to normal individuals [21].

However, discrepancies in the abundance changes across different studies remain. Wu et al. found that in AML patients, the relative abundance of *Firmicutes* was lower compared to healthy controls (54% vs. 71%), while *Bacteroidetes* abundance increased (35% vs. 19%) [22]. Subsequent research by Wang et al. further confirmed that AML patients exhibited not only a decreased relative abundance of *Firmicutes*, but also an increased relative abundance of *Bacteroidota*, accompanied by a reduction in *Faecalibacterium*, *Roseburia*, *Subdoligranulum*, and *Bifidobacterium* [15].

In addition to these core microbiota changes, recent studies have unveiled more complex mechanisms of microbiota remodeling. Pötgens et al. used 16S rRNA high-throughput sequencing and found that in AML patients, *Blautia* and *Parabacteroides* were abnormally proliferated, while the abundance of *Eubacterium eligens* was reduced by over threefold [23]. Of particular note are significant changes in the relative abundances of gut bacteria associated with metabolic dysregulation (e.g., *Blautia*, *Prevotella*, *Phenylacetate*, and *Hippurate*). Yu et al. demonstrated that compared to healthy controls, AML patients showed an increase in the relative abundance of *Acidobacteria* and *Chloroflexi* at the phylum level, and a decrease in *Tenericutes*. At the genus level, *Streptococcus* abundance decreased, while *Megamonas*, *Lachnospiraceae NC2004 group*, and *Prevotella_9* showed increased relative abundance [24]. Additionally, compared to healthy individuals, AML patients’ gut microbiota were enriched in the genera *Sphingomonas*, *Lysobacter*, *Helicobacter*, *Lactobacillus*, and *Enterococcus*.

Integrating the existing research evidence, AML-associated gut microbiota dysbiosis manifests as a multi-level ecological disturbance. At the phylum level, the *Firmicutes*/*Bacteroidetes* ratio shows dynamic imbalance [20,22], with *Acidobacteria* and *Chloroflexi* showing increased relative abundance, while *Tenericutes* abundance decreases [24]. At the genus/family level, the abnormal enrichment of gut microbes such as *Megamonas*, *Lachnospiraceae NC2004 group*, and *Prevotella 9*, along with the consistent depletion of *Streptococcus*, strongly suggests their potential as diagnostic biomarkers and therapeutic targets.

### 3.2. Gut Microbial Metabolic Products

Recent studies indicate that gut microbiota metabolic products can regulate AML progression through the “gut-bone marrow axis.” Wang et al. employed gas chromatography-mass spectrometry (GC-MS) to systematically reveal the metabolic characteristics of AML in a mouse model [15]. The gut microbiota-derived metabolite butyrate enhances the intestinal physical barrier by promoting tight junction protein synthesis, strengthens the chemical barrier via stimulating mucin secretion, and modulates the immune barrier by suppressing inflammatory responses and promoting T cell differentiation. Additionally, butyrate supports energy provision and activates relevant signaling pathways to facilitate the repair and regeneration of intestinal epithelial cells [25]. Compared to healthy controls, the concentration of butyrate in the colon contents of AML mice was significantly reduced, leading to compromised intestinal barrier integrity. This metabolic dysregulation facilitates the entry of LPS through the impaired gut barrier into the bloodstream, exacerbating the progression of AML in mice [15]. This suggests that the butyrate-LPS axis could serve as an important biomarker combination for AML diagnosis and treatment. Notably, in addition to the butyrate system, amino acids serve as the fundamental building blocks for protein synthesis, while their derivatives function as crucial sources of energy and nucleotide precursors, play pivotal roles in maintaining redox homeostasis, and contribute significantly to physiological equilibrium, epigenetic regulation, and protein modulation. Notably, neoplastic cells exhibit distinct patterns in the relative production, uptake, and downstream utilization of amino acids compared to their healthy counterparts [26]. And amino acid metabolism also plays a crucial role in AML pathology. Wu et al. demonstrated via UHPLC-Qexact analysis that serum concentrations of carnosine were significantly elevated in AML patients compared to controls, along with a concurrent increase in its downstream metabolite L-histidine [22]. Further studies revealed that the microbiota-host co-metabolic network plays an essential regulatory role in AML development. Xu et al. used multi-omics integration to demonstrate that the relative abundances of *Collinsella* and *Coriobacteriaceae* in the feces of AML patients were positively correlated with serum concentrations of hydroxypropionyl-hydroxyproline and prolyl-tyrosine [20] (Table 2).

Liu et al. further investigated how bile acids, a class of gut microbial metabolites, affect AML progression. Their research revealed that bile acids regulate AML by promoting lipid droplet accumulation, inducing lipid peroxidation, and inhibiting the polarization of M2 macrophages [17]. Using LC-MS, they found that CDCA levels were significantly reduced in the feces and plasma of AML patients. Further analysis showed that CDCA levels were positively correlated with gut microbiota diversity, while inversely correlating with poor prognosis in AML patients.

These findings highlight the potential clinical value of gut microbiota metabolic products in AML diagnosis and targeted therapy. Gut microbiota, through its metabolic activity—particularly SCFAs, bile acids and other metabolites—can regulate immune responses, impact mitochondrial morphology and function, and maintain gut barrier integrity. These mechanisms offer promising new biomarkers and therapeutic targets for the early diagnosis and treatment of AML. Furthermore, the metabolic products of gut microbiota show considerable potential in modulating AML cell growth, overcoming chemotherapy resistance, and enhancing treatment responses. Therefore, gut microbiota and its metabolic products could serve not only as supportive tools in AML diagnosis but also as novel strategies for targeted therapy, paving the way for personalized medicine and precision treatments.

## 4. Prognosis and Intervention

### 4.1. Gut Microbiota in Prognosis of AML

The prognosis of AML is significantly influenced by both the intestinal barrier function and the development of chemotherapy resistance. A growing body of evidence suggests that chemotherapy not only alters the composition of the gut microbiota but also impacts its metabolic activity, which in turn can affect treatment responses and clinical outcomes (Table 3).

Recent studies have highlighted that after undergoing treatments such as chemotherapy or HSCT, AML patients experience notable changes in their gut microbiota and its metabolic products, which correlate with patient prognosis. Specifically, following induction chemotherapy, there is a decrease in microbiota diversity in AML patients, which is marked by an increase in the relative abundance of taxa such as *Lactobacillaceae*, *Campylobacter*, *Enterococcus faecium*, and *Staphylococcus*. Simultaneously, key metabolic products—such as urinary hippurate and fecal bacterial amino acid metabolites (including 2-methylbutyrate, isovalerate, and phenylacetate)—are significantly reduced, leading to compromised gut barrier function [16]. Furthermore, these alterations in the gut microbiota have been strongly associated with cachexia-related features, including appetite loss, weight loss, and muscle wasting. Similarly, reduced microbiota diversity following chemotherapy and HSCT has been linked to an increase in transplant-related mortality rates [27]. These findings underscore the potential therapeutic benefit of strategies aimed at protecting gut barrier integrity and modulating the gut microbiota to improve AML treatment outcomes.

Xu et al. further observed that post-chemotherapy, there was an increase in the Chao1 index of gut microbiota diversity, with a notable enrichment of *Actinobacteria*, *Bifidobacterium*, and *Bifidobacteriales*. This was accompanied by significant changes in the metabolic profiles of amino acids and their analogs [20]. Additionally, during the neutropenic phase, *Enterococcus* abundance increased. At the onset of neutropenia, the relative abundance of *Firmicutes* rose from 34.3% to 50.8%, only to decrease again following bone marrow recovery [21]. Notably, the administration of tigecycline has been shown to disrupt gut microbiota composition, which correlates closely with the development of chemotherapy resistance and poorer prognostic outcomes in high-risk patients [28].

Protecting gut barrier integrity during treatment has emerged as a key strategy for improving patient prognosis. In studies by Hueso et al., Tg222 mice, which overexpress a mucosal protease to enhance the mucus layer, exhibited elevated citrulline levels during chemotherapy, promoting epithelial repair [29]. This study emphasizes that enhancing gut barrier function may help reduce bloodstream infections and improve the prognosis of AML patients. Similarly, research by Liu et al. demonstrated that curcumin can modulate the gut microbiota in AML mice, decreasing the abundance of mucus-degrading bacteria and thus increasing the sensitivity of the mice to cytarabine [30]. These findings further confirm the critical role of gut barrier integrity in overcoming chemotherapy resistance.

Clinical studies provide additional support for these findings. Renga et al. found that CPX-351 therapy protected AML patients from gut microbiota dysbiosis and intestinal mucosal damage. This treatment suppressed intestinal inflammation, reduced epithelial permeability, and enhanced survival by activating the Aryl hydrocarbon receptor (AhR)-IL-22-IL-10 signaling pathway and modulating anaerobic bacterial metabolites [31]. Moreover, the use of tigecycline after chemotherapy has also been shown to significantly alter the gut microbiota composition in AML patients [28].

Interestingly, the pre-treatment gut microbiota characteristics may serve as important prognostic biomarkers for treatment response. Studies utilizing 16S rRNA sequencing have shown that higher Shannon diversity and increased abundance of the *Porphyromonadaceae* family are associated with a reduced risk of infection during the neutropenic phase [32]. Furthermore, early recovery of absolute lymphocyte count (ALC) during treatment is one of the strongest prognostic factors, with patients demonstrating complete recovery typically exhibiting higher α diversity in their gut microbiota compared to those with incomplete recovery [33].

The dynamic changes in the gut microbiota of AML patients are closely linked to chemotherapy efficacy and prognosis. Chemotherapy induces a reduction in microbiota diversity, promotes the proliferation of opportunistic pathogens, and compromises the gut barrier, all of which contribute to increased cachexia and infection risk. Strategies aimed at protecting gut barrier integrity and regulating microbiota composition can enhance chemotherapy sensitivity and improve survival outcomes. Additionally, baseline microbiota diversity and the abundance of specific bacterial families, such as *Porphyromonadaceae*, could serve as predictive biomarkers for treatment response. Targeted development of gut microbiota modulation strategies as adjunctive treatments for AML is a promising avenue for future research.

In conclusion, gut microbiota dysbiosis, intestinal barrier dysfunction, and metabolic disturbances are critical contributors to chemotherapy resistance and poor prognosis in AML. Future research should focus on exploring gut microbiota-based interventions as a means of optimizing AML treatment regimens and improving patient survival.

**Table 3:** Summary of studies related to the gut microbiota-targeted therapeutic interventions on AML.

Class	Study Subjects	Therapeutic Interventions	Intervention-Associated Alterations	Reference
Population Studies	AML patients	Intensive chemotherapy	AML patients exhibit decreased appetite, weight loss, and muscle wasting during hospitalization, with a 50% incidence of malignant disease. Intensive chemotherapy of AML temporarily damages the intestinal barrier function of patients, leading to changes in the gut microbiota, with a decrease in diversity and an increase in the relative abundance of Lactobacillaceae, Campylobacter, Enterococcus faecium, Staphylococcus. Reduction in urinary hippurate and fecal bacterial amino acid metabolites (bAAm) (2-methylbutyrate, isovalerate, phenylacetate).	[16]
	AML patients	Chemotherapy	Observed species index increase in Chao1 index in patients after chemotherapy treatment. Actinobacteria, Bifidobacterium, Bifidobacteriales intestinalis enrichment in patients after chemotherapy treatment. Many differential amino acids and analogs observed in AML patients treated with chemotherapy.	[20]
	AML patients	Induction chemotherapy	Enterococcus was more abundant in the neutropenic phase, and Escherichianotably declined compared to pretreatment. Firmicutes predominate on the first day of febrile neutropenia after chemotherapy, with relative abundance increasing from 34.3% to 50.8% and then declining after bone marrow recovery.	[21]
	AML patients	Stem cell transplant	Reduced gut microbiota diversity with chemotherapy, and stem cell transplantation is associated with higher mortality after transplantation. After hospital discharge, the trajectory of gut microbiota recovery in AML patients produces a new community that is highly different from baseline.	[27]
	AML patients	Post-chemotherapy tigecycline therapy	Tigecycline treatment significantly alters the distribution of intestinal flora in AML patients, and these changes in intestinal flora have been associated with chemotherapy resistance. Long-term use of antibiotics not only leads to ecological dysregulation of the intestinal flora but also indirectly affects the sensitivity of chemotherapeutic drugs. RNA sequencing was performed on CHO cells, and the expression of most genes was downregulated after tigecycline treatment. Survival results showed that high expression of these genes was associated with poorer prognosis. There was no significant difference in the alpha diversity of the intestinal flora of the patients before and after tigecycline treatment, and β diversity analysis showed significant differences between the intestinal flora of both groups. Based on the genetically constructed risk model of tigecycline-regulated genes, and by analyzing multiple databases and inflammation, this study found that patients with high-risk population characteristics had a significantly worse prognosis.	[28]
	AML patients, WT mice	Intensive chemotherapy	WT mice have decreased levels of citrulline and increased *E. coli* and *Enterococcus* spp. loads with histological damage during chemotherapy, whereas Tg222 mice (which release histones that enhance the mucus layer) have higher levels of citrulline and are able to heal epithelium more rapidly after chemotherapy. Strengthening the intestinal barrier is a promising approach to limit bloodstream infections and improve the prognosis of patients with AML.	[29]
	AML patients	CPX-352	Improved survival and prognosis of patients undergoing hematopoietic stem cell transplantation. Protecting patients from ecological dysbiosis of the gut microbiota, intestinal mucosal damage, and intestinal diseases while increasing resistance to antifungal drugs. Protective effects on the host are mainly through activation of the aromatic hydrocarbon receptor-IL-22-IL-10 host pathway and production of immunomodulatory metabolites by anaerobic bacteria. CPX-352 treatment not only did not actively promote the progression of intestinal pathology but also inhibited ongoing intestinal injury, attenuating the inflammatory response and the increase in intestinal epithelial permeability. CPX-351 activates the protective AhR pathway. CPX-351 promotes transplantable protective microbial communities. CPX-351 does not induce ecological dysregulation of gut microbiota. CPX-351 promotes microbial metabolites and colonization resistance to fungi.	[31]
	AML patients	Induction Therapy	Higher Shannon diversity and higher relative abundance of Porphyromonadaceae in the gut microbiota prior to induction chemotherapy are associated with an increased probability of remaining infection-free during neutropenia. Patients treated with carbapenems for >72 h had significantly lower alpha diversity at neutrophil recovery and were approximately 4-fold more likely to be infected within 90 d of neutrophil recovery.	[32]
	AML patients	Induction therapy, subcutaneous granulocyte colony-stimulating factor therapy, intensive chemotherapy	The patient’s absolute lymphocyte count (ALC) recovered to 500/mm^3^, and early ALC recovery was considered the strongest prognostic factor. Fully recovered patients tend to have higher α diversity than incompletely recovered patients. At the family level, patients with complete blood recovery showed an increase in the relative abundance of Odoribacteraceae, Barnesiellaceae, and Alcaligenaceae and a decrease in the relative abundance of Micrococcaceae and Enterococcaceae; at the genus level, there are higher proportions of Blautia, Butyricimonas, Eubacterium, Odoribacter, Anaerotruncus, Dorea, Sutterella, and Ruminococcus and lower proportions of Rothia and Enterococcus.	[33]
Animal Experiment	AML mice	Curcumin	Curcumin treatment alters gut flora in AML mice and contributes to sensitization to cytarabine. Enhancement of intestinal sensitization to cytarabine in AML mice by reducing mucus-degrading bacteria. Curcumin treatment inhibits cholesterol synthesis and increases sensitivity to acitretin. Curcumin sensitizes the response to cytarabine by modulating gut microbiota, highlighting the importance of intestinal integrity fortification in the treatment of chemotherapy resistance.	[30]

### 4.2. Gut Microbiota in the Intervention of AML

In recent years, as our understanding of the relationship between the gut microbiota and host health deepens, microbiota modulation has emerged as an innovative therapeutic approach in oncology. This is particularly relevant in the treatment of AML, where an increasing number of studies suggest that the composition of the gut microbiota and its metabolites significantly influence the onset, progression, and treatment response of AML. Among the various microbiota modulation strategies, fecal microbiota transplantation (FMT) has emerged as the most prominent approach in clinical practice. FMT has shown considerable potential in improving AML treatment outcomes, alleviating chemotherapy side effects, and optimizing clinical prognosis (Table 3).

FMT is a more direct method of gut microbiota intervention, which involves transplanting the gut microbiota of a healthy donor into the patient’s intestinal tract. This procedure aims to rebuild and restore microbiota diversity and function. The core mechanism behind this therapeutic approach lies in the introduction of a healthy, functional microbiota from the donor, which restores the diversity and metabolic homeostasis of the host’s gut microbiota [34]. Recent studies have increasingly focused on the pivotal role of FMT in AML therapy, particularly in its ability to reshape the gut microbiota structure, such as increasing short-chain fatty acid-producing bacteria, restore microbiota diversity, and reduce leukemia burden.

Recent research indicates that microbiota regulation has potential therapeutic value in the treatment of hematological malignancies. Wang et al. investigated the therapeutic effects and mechanisms of FMT in an AML mouse model, systematically evaluating its impact through various indices [15]. The experimental design employed an oral gavage method for allogeneic microbiota transplantation. Through multidimensional assessments, it was found that the FMT-treated mice exhibited a significantly smaller spleen size and lower organ weight (*p* < 0.001) compared to the control group, with reduced leukemia cell infiltration. To elucidate the underlying mechanisms, the researchers employed 16S rRNA high-throughput sequencing to monitor dynamic changes in the gut microbiota. The results revealed that the α diversity index of the gut microbiota in AML-induced mice was lower compared to the normal group, but this was reversed following FMT intervention. Notably, when *Escherichia coli* strains were added to the standard FMT protocol, the leukemia burden was further reduced, suggesting a synergistic effect of specific functional microbiota.

In clinical translation, a randomized controlled trial has confirmed the clinical potential of FMT [35]. Compared to the placebo group, the FMT group of patients showed a reduction in the incidence and density of invasive infections within 4 months post-transplantation, with a 25–30% colonization rate of donor-derived microbiota. Metagenomic analyses revealed a significant reduction in the relative abundance of gut bacteria such as *Enterococcus*, *Streptococcus*, and *Veillonella* in the FMT group, aligning closely with microbial reshaping patterns reported by Malard et al. One key finding was the unique advantage of autologous FMT (AFMT). Experimental data demonstrated that AFMT treatment restored the α diversity index of the gut microbiota in AML mice to a state similar to baseline. Based on species-level richness and diversity indices, the results suggested a successful reconstruction of the microbiota in the mice [36].

Translating FMT into clinical practice for AML faces significant challenges. Foremost is the safety risk in profoundly immunocompromised patients. Stringent donor screening and advanced pathogen detection are essential to mitigate transmission of pathogens or opportunistic organisms, which escalates complexity and cost [37]. Equally problematic is the absence of standardized protocols [38]. Substantial variations exist in donor criteria, stool processing, administration routes, dosing, and treatment frequency. Such heterogeneity complicates cross-trial comparisons and prevents establishment of reliably effective AML regimens. Furthermore, the long-term stability of engrafted microbiota also remains unestablished [39].

FMT shows significant promise in AML treatment by reshaping gut microbiota structure, restoring microbial diversity, and maintaining metabolic homeostasis. Research indicates that FMT can reduce leukemia burden, lower chemotherapy-associated infections, and improve prognosis by increasing the abundance of short-chain fatty acid-producing bacteria. Both clinical and animal studies have confirmed that FMT can effectively regulate pathogenic bacteria abundance, such as *Enterococcus*, with AFMT proving particularly beneficial for microbiota reconstruction. Additionally, the supplementation of specific functional strains (e.g., *Escherichia coli*) can have a synergistic effect. Future research should focus on optimizing donor screening and microbiota transplantation strategies to enhance the clinical translation of gut microbiota-based interventions in AML therapy.

## 5. Perspectives and Conclusions

The gut microbiota and its metabolites play crucial roles in the pathogenesis, therapeutic response, and prognosis of AML, offering potential biomarkers for diagnosis and prognostic evaluation.

However, translating the findings from gut microbiota research into clinical applications remains a significant challenge. This includes the need for personalized interventions and the spatiotemporal heterogeneity of fecal samples. One of the primary challenges in gut microbiota research is the issue of individualized microbiota interventions. The host’s genetic background can substantially influence the composition of the gut microbiota and its response to interventions. Future research must consider the impact of host genetic factors on the effectiveness of microbiota modulation and integrate multi-omics data, such as genomics, gut microbiomics, and metabolomics, to develop more precise predictive models. These models could not only predict the efficacy of microbiota interventions but also assist in designing personalized treatment regimens, thereby enhancing the precision and effectiveness of AML therapies.

The spatiotemporal heterogeneity of fecal samples represents another pressing challenge. Since the composition of the gut microbiota is significantly influenced by factors such as circadian rhythms, diet, and antibiotic usage, samples collected at different time points may exhibit considerable variability, posing a challenge to the consistency of microbiota detection. Thus, the development of standardized sampling and analytical protocols is critical to addressing this issue. For example, establishing standardized sampling times, processing conditions, and storage methods would enhance the comparability and reproducibility of microbiota data. Additionally, the instability of gut microbiota in healthy donor populations further complicates the consistency of therapeutic outcomes, highlighting the need for rigorous donor selection criteria.

In conclusion, the gut microbiota demonstrates tremendous potential in the diagnosis, treatment, and prognostic evaluation of AML. The future of AML therapy will likely be shaped by microbiota-host interaction-based precision medicine, opening new avenues for comprehensive treatment strategies. Continued research in this field will be pivotal in overcoming current challenges and moving towards more effective, personalized interventions in AML.

## Figures and Tables

**Table 1:** Summary of studies related to the gut microbiota compositional alterations in AML.

Study Subjects	Compositional Changes in Gut Microbiota	Biomarker Gut Microbiota	Reference
AML patients, WT mice	Significantly lower gut microbiota diversity in AML patients compared to healthy control populations. At the gate level, AML patients had significantly lower relative abundance of *Firmicutes* and higher relative abundance of *Bacteroidota*. At the genus level, AML patients had increased relative abundance of *Bacteroides* and decreased relative abundance of *Faecalibacterium*, *Roseburia*, *Subdoligranulum*, and *Bifidobacterium*. Relative abundance of *Faecalibacterium* and *Roseburia* in feces is lower in AML patients compared to controls.	Low diversity of gut microbiota and reduced *Faecalibacterium* play an important role in the progression of AML.	[15]
AML patients	The gut microbiota community is relatively small in AML patients compared to the control population. Increased proportion of *Firmicutes* to *Bacteroidetes* in AML patients compared to the normal population. LEfSe analysis identifies *Collinsella* and *Coriobacteriaceae* as biomarkers in AML patients.	*Firmicutes*, *Bacteroidetes*, *Collinsella*, *Coriobacteriaceae*	[20]
AML patients	*Firmicutes* prevailed.	*Firmicutes*	[21]
AML model mice, AML patients	Decrease in relative abundance of *Firmicutes*, increase in relative abundance of *Bacteroidetes*. The dominant bacteria in AML patients were *Firmicutes* (54%) and *Bacteroidetes* (35%); the dominant bacteria in controls were *Firmicutes* (71%) and *Bacteroidetes* (19%).	*Firmicutes*, *Bacteroidetes*	[22]
AML patients	Threefold depletion of *Eubacterium eligens* abundance strongly correlates with reduced muscle strength and diminished citrulline levels (a biomarker of enterocyte integrity and functionality). Enriched colonization of *Blautia* and *Parabacteroides genera* exhibits significant association with anorexia-related pathophysiology. Specific bacterial taxa (*Blautia*, *Prevotella*) and metabolomic signatures (phenylacetate, hippurate) demonstrate significant alterations in relative abundance correlating with glycemic dysregulation.	*Eubacterium eligens*, *Blautia*, *Parabacteroides*, *Prevotella*, *Phenylacetate*, and *Hippurate*	[23]
AML patients	AML patients and healthy control populations show significant differences in gut microbiota α diversity and β diversity. At the portal level, the relative abundance of *Acidobacteria* and *Chloroflexi* was increased, and the relative abundance of *Tenericutes* was decreased in the gut microbiota of AML patients. At the genus level, *Streptococcus* decreased, and Megamonas, LachnospiraceaeNC2004 group, and Prevotella 9 increased in relative abundance. At the genus level, *Streptococcus* decreased, and *Megamonas*, *LachnospiraceaeNC2004* group, and *Prevotella 9* increased in relative abundance.	*Actinobacteria*, *Acidobacteria*, *Chloroflexi*, *Tenericutes*, *Streptococcus*, *Megamonas*, *LachnospiraceaeNC2004 group*, *Prevotella 9*, *Sphingomonas*, *Lysobacyer*, *Helicobacter*, *Lactobacillus*, *Enterococcus*	[24]

**Table 2:** Summary of studies related to gut microbiota-derived metabolite alterations in AML.

Class	Study Subjects	Alterations in Gut Microbiota-Derived Metabolites	Biomarker Metabolites	Reference
Population Studies	AML patients, Murine AML cell line, WT mice	Decreased levels of butyrate produced by the patient’s gut microbiota. Impaired intestinal barrier in mice accelerates leakage of LPS into the bloodstream. Significantly reduced levels of propionate and butyrate, metabolites of gut microbiota, in AML patients. *Faecalibacterium* and butyric acid were positively correlated, and the relative abundance of *Faecalibacterium* was significantly down-regulated, proving to be closely associated with butyric acid metabolism. Cellular assays demonstrate that butyrate is a direct metabolite of *Faecalibacterium* and that *Faecalibacterium* can cause an increase in butyrate content. LPS can enter the bloodstream through a damaged intestinal barrier, exacerbating the progression of AML.	LPS, butyrate	[15]
	AML patients, Human AML cell line	Reduced CDCA in feces and plasma of AML patients shows a positive correlation with intestinal flora diversity and a negative correlation with AML prognosis	CDCA	[17]
	AML patients	Bacterial biomarkers *Collinsella* and *Coriobacteriaceae* are significantly and positively correlated with hydroxypropionyl-hydroxyproline, prolyl-tyrosine and tyrosyl-proline in AML patients.	hydroxypropionyl-hydroxyproline, prolyl-tyrosine and tyrosyl-proline	[20]
	AML patients	Significant difference in metabolites between patients and controls Significant negative correlation between the key metabolite myostatin and *Peptococcaceae* and *Campylobacteraceae*. There were 11 significantly different metabolic pathways between the two groups: amino acid metabolism, vitamin metabolism and carbohydrate metabolism, polysaccharide metabolism. Decreased levels of luteolinic acid, pyridoxamine, indole and 5-methylcytosine and increased levels of L-histidine, N-acetyl-L-methionine and gluconolactone in the feces of mice in the AML group. Decreased serum levels of cytidine and caprylic acid and increased levels of glucose 6-phosphate, L-carnitine, leucine, L-glutamic acid, N-hydroxyglutamic acid neuraminic acid, glucose 6-phosphate, and myostatin. L-histidine levels in the feces were increased in the AML group and returned to normal after treatment, suggesting that the myostatin-histidine pathway may play a role in the development of AML, since histidine is a downstream metabolite of the myostatin metabolic pathway.	L-histidine, myostatin	[22]

## Data Availability

No new data were created or analyzed in this study.

## References

[1] Lagunas-Rangel F.A., Chávez-Valencia V., Gómez-Guijosa M.Á., Cortes-Penagos C. (2017). Acute Myeloid Leukemia-Genetic Alterations and Their Clinical Prognosis. Int. J. Hematol. Oncol. Stem Cell Res..

[2] Sasaki K., Ravandi F., Kadia T.M., DiNardo C.D., Short N.J., Borthakur G., Jabbour E., Kantarjian H.M. (2021). De novo acute myeloid leukemia: A population-based study of outcome in the United States based on the Surveillance, Epidemiology, and End Results (SEER) database, 1980 to 2017. Cancer.

[3] Lachowiez C.A., Loghavi S., Kadia T.M., Daver N., Borthakur G., Pemmaraju N., Naqvi K., Alvarado Y., Yilmaz M., Short N. (2020). Outcomes of older patients with NPM1-mutated AML: Current treatments and the promise of venetoclax-based regimens. Blood Adv..

[4] Mims A.S., Kohlschmidt J., Borate U., Blachly J.S., Orwick S., Eisfeld A.-K., Papaioannou D., Nicolet D., Mrόzek K., Stein E. (2021). A precision medicine classification for treatment of acute myeloid leukemia in older patients. J. Hematol. Oncol..

[5] Bennett J.M., Catovsky D., Daniel M.T., Flandrin G., Galton D.A., Gralnick H.R., Sultan C. (1976). Proposals for the classification of the acute leukaemias. French-American-British (FAB) co-operative group. Br. J. Haematol..

[6] Khoury J.D., Solary E., Abla O., Akkari Y., Alaggio R., Apperley J.F., Bejar R., Berti E., Busque L., Chan J.K.C. (2022). The 5th edition of the World Health Organization Classification of Haematolymphoid Tumours: Myeloid and Histiocytic/Dendritic Neoplasms. Leukemia.

[7] Döhner H., Wei A.H., Appelbaum F.R., Craddock C., DiNardo C.D., Dombret H., Ebert B.L., Fenaux P., Godley L.A., Hasserjian R.P. (2022). Diagnosis and management of AML in adults: 2022 recommendations from an international expert panel on behalf of the ELN. Blood.

[8] Eisfeld A.-K., Mardis E.R. (2024). Acute Myeloid Leukemia Genomics: Impact on Care and Remaining Challenges. Clin. Chem..

[9] Awada H., Mustafa Ali M.K., Thapa B., Awada H., Seymour L., Liu L., Gurnari C., Kishtagari A., Wang E., Baer M.R. (2022). A Focus on Intermediate-Risk Acute Myeloid Leukemia: Sub-Classification Updates and Therapeutic Challenges. Cancers.

[10] Sallman D.A., McLemore A.F., Aldrich A.L., Komrokji R.S., McGraw K.L., Dhawan A., Geyer S., Hou H.-A., Eksioglu E.A., Sullivan A. (2020). TP53 mutations in myelodysplastic syndromes and secondary AML confer an immunosuppressive phenotype. Blood.

[11] Zhang J., Gu Y., Chen B. (2019). Mechanisms of drug resistance in acute myeloid leukemia. Onco Targets Ther..

[12] Takiishi T., Fenero C.I.M., Câmara N.O.S. (2017). Intestinal barrier and gut microbiota: Shaping our immune responses throughout life. Tissue Barriers.

[13] Fan Y., Pedersen O. (2021). Gut microbiota in human metabolic health and disease. Nat. Rev. Microbiol..

[14] Qiao S., Liu C., Sun L., Wang T., Dai H., Wang K., Bao L., Li H., Wang W., Liu S.-J. (2022). Gut Parabacteroides merdae protects against cardiovascular damage by enhancing branched-chain amino acid catabolism. Nat. Metab..

[15] Wang R., Yang X., Liu J., Zhong F., Zhang C., Chen Y., Sun T., Ji C., Ma D. (2022). Gut microbiota regulates acute myeloid leukaemia via alteration of intestinal barrier function mediated by butyrate. Nat. Commun..

[16] Pötgens S.A., Lecop S., Havelange V., Li F., Neyrinck A.M., Neveux N., Maertens J., Walter J., Schoemans H., Delzenne N.M. (2023). Gut microbiota alterations induced by intensive chemotherapy in acute myeloid leukaemia patients are associated with gut barrier dysfunction and body weight loss. Clin. Nutr..

[17] Liu J., Wei Y., Jia W., Can C., Wang R., Yang X., Gu C., Liu F., Ji C., Ma D. (2022). Chenodeoxycholic acid suppresses AML progression through promoting lipid peroxidation via ROS/p38 MAPK/DGAT1 pathway and inhibiting M2 macrophage polarization. Redox Biol..

[18] Wei Y., Liu W., Wang R., Chen Y., Liu J., Guo X., Can C., Yang X., Wang D., Hu X. (2024). Propionate promotes ferroptosis and apoptosis through mitophagy and ACSL4-mediated ferroptosis elicits anti-leukemia immunity. Free Radic. Biol. Med..

[19] de Martel C., Ferlay J., Franceschi S., Vignat J., Bray F., Forman D., Plummer M. (2012). Global burden of cancers attributable to infections in 2008: A review and synthetic analysis. Lancet Oncol..

[20] Xu J., Kang Y., Zhong Y., Ye W., Sheng T., Wang Q., Zheng J., Yang Q., Yi P., Li Z. (2023). Alteration of gut microbiome and correlated amino acid metabolism are associated with acute myelocytic leukemia carcinogenesis. Cancer Med..

[21] Rattanathammethee T., Tuitemwong P., Thiennimitr P., Sarichai P., Na Pombejra S., Piriyakhuntorn P., Hantrakool S., Chai-Adisaksopha C., Rattarittamrong E., Tantiworawit A. (2020). Gut microbiota profiles of treatment-naïve adult acute myeloid leukemia patients with neutropenic fever during intensive chemotherapy. PLoS ONE.

[22] Wu B., Xu Y., Tang M., Jiang Y., Zhang T., Huang L., Wang S., Hu Y., Zhou K., Zhang X. (2023). A Metabolome and Microbiome Analysis of Acute Myeloid Leukemia: Insights into the Carnosine-Histidine Metabolic Pathway. Toxics.

[23] Pötgens S.A., Havelange V., Lecop S., Li F., Neyrinck A.M., Bindels F., Neveux N., Demoulin J.-B., Moors I., Kerre T. (2024). Gut microbiome alterations at acute myeloid leukemia diagnosis are associated with muscle weakness and anorexia. Haematologica.

[24] Yu D., Yu X., Ye A., Xu C., Li X., Geng W., Zhu L. (2021). Profiling of gut microbial dysbiosis in adults with myeloid leukemia. FEBS Open Bio.

[25] Recharla N., Geesala R., Shi X.-Z. (2023). Gut Microbial Metabolite Butyrate and Its Therapeutic Role in Inflammatory Bowel Disease: A Literature Review. Nutrients.

[26] Mishra S.K., Millman S.E., Zhang L. (2023). Metabolism in acute myeloid leukemia: Mechanistic insights and therapeutic targets. Blood.

[27] Rashidi A., Ebadi M., Rehman T.U., Elhusseini H., Halaweish H.F., Kaiser T., Holtan S.G., Khoruts A., Weisdorf D.J., Staley C. (2022). Lasting shift in the gut microbiota in patients with acute myeloid leukemia. Blood Adv..

[28] Zhong F.-L., He J.-J., Bai K.-H., Shao R.-N., Wu G.-Y., Tian X.-P., Wang D.-W., Dai Y.-J., Chen S.-L. (2024). Tigecycline-induced coagulation gene prognostic prediction model and intestinal flora signature in AML. Front. Immunol..

[29] Hueso T., Ekpe K., Mayeur C., Gatse A., Joncquel-Chevallier Curt M., Gricourt G., Rodriguez C., Burdet C., Ulmann G., Neut C. (2020). Impact and consequences of intensive chemotherapy on intestinal barrier and microbiota in acute myeloid leukemia: The role of mucosal strengthening. Gut Microbes.

[30] Liu J., Luo W., Chen Q., Chen X., Zhou G., Sun H. (2022). Curcumin sensitizes response to cytarabine in acute myeloid leukemia by regulating intestinal microbiota. Cancer Chemother. Pharmacol..

[31] Renga G., Nunzi E., Stincardini C., Pariano M., Puccetti M., Pieraccini G., Di Serio C., Fraziano M., Poerio N., Oikonomou V. (2024). CPX-351 exploits the gut microbiota to promote mucosal barrier function, colonization resistance, and immune homeostasis. Blood.

[32] Galloway-Peña J.R., Shi Y., Peterson C.B., Sahasrabhojane P., Gopalakrishnan V., Brumlow C.E., Daver N.G., Alfayez M., Boddu P.C., Khan M.A.W. (2020). Gut Microbiome Signatures Are Predictive of Infectious Risk Following Induction Therapy for Acute Myeloid Leukemia. Clin. Infect. Dis..

[33] Salvestrini V., Conti G., D’Amico F., Cristiano G., Candela M., Cavo M., Turroni S., Curti A. (2025). Gut Microbiome as a Potential Marker of Hematologic Recovery Following Induction Therapy in Acute Myeloid Leukemia Patients. Cancer Med..

[34] Yadegar A., Bar-Yoseph H., Monaghan T.M., Pakpour S., Severino A., Kuijper E.J., Smits W.K., Terveer E.M., Neupane S., Nabavi-Rad A. (2024). Fecal microbiota transplantation: Current challenges and future landscapes. Clin. Microbiol. Rev..

[35] Rashidi A., Ebadi M., Rehman T.U., Elhusseini H., Kazadi D., Halaweish H., Khan M.H., Hoeschen A., Cao Q., Luo X. (2023). Randomized Double-Blind Phase II Trial of Fecal Microbiota Transplantation Versus Placebo in Allogeneic Hematopoietic Cell Transplantation and AML. J. Clin. Oncol..

[36] Malard F., Vekhoff A., Lapusan S., Isnard F., D’Incan-Corda E., Rey J., Saillard C., Thomas X., Ducastelle-Lepretre S., Paubelle E. (2021). Gut microbiota diversity after autologous fecal microbiota transfer in acute myeloid leukemia patients. Nat. Commun..

[37] Zhang X., Ishikawa D., Nomura K., Fukuda N., Haraikawa M., Haga K., Shibuya T., Mita T., Nagahara A. (2022). Donor Screening Revisions of Fecal Microbiota Transplantation in Patients with Ulcerative Colitis. J. Clin. Med..

[38] Lopetuso L.R., Deleu S., Godny L., Petito V., Puca P., Facciotti F., Sokol H., Ianiro G., Masucci L., Abreu M. (2023). The first international Rome consensus conference on gut microbiota and faecal microbiota transplantation in inflammatory bowel disease. Gut.

[39] Nooij S., Ducarmon Q.R., Laros J.F.J., Zwittink R.D., Norman J.M., Smits W.K., Verspaget H.W., Keller J.J., Terveer E.M., Kuijper E.J. (2021). Fecal Microbiota Transplantation Influences Procarcinogenic Escherichia coli in Recipient Recurrent Clostridioides difficile Patients. Gastroenterology.

